# Acid-Responsive Adamantane-Cored Amphiphilic Block Polymers as Platforms for Drug Delivery

**DOI:** 10.3390/nano11010188

**Published:** 2021-01-13

**Authors:** Weiqiu Wen, Chong Guo, Jianwei Guo

**Affiliations:** 1 School of Chemical Engineering & Light Industry, Guangdong University of Technology, Guangzhou 510006, China; will_wenwq@163.com; 2 College of Chemistry and Chemical Engineering, Shanghai University of Engineering Science, Shanghai 201620, China; gcsues@163.com

**Keywords:** acid-responsive, polymeric micelles, drug delivery, controlled release, dissipative particle dynamics simulation

## Abstract

Four-arm star-shaped (denoted as ‘S’) polymer adamantane-[poly(lactic-*co*-glycolic acid)-*b*-poly(*N,N’*-diethylaminoethyl methacrylate) poly(ethylene glycol) monomethyl ether]_4_ (S-PLGA-D-P) and its linear (denoted as ‘L’) counterpart (L-PLGA-D-P) were synthesized, then their self-assembled micelles were further developed to be platforms for anticancer drug delivery. Two types of polymeric micelles exhibited strong pH-responsiveness and good drug loading capacity (21.6% for S-PLGA-D-P and 22.9% for L-PLGA-D-P). Using doxorubicin (DOX) as the model drug, their DOX-loaded micelles displayed well controlled drug release behavior (18.5–19.0% of DOX release at pH 7.4 and 77.6–78.8% of DOX release at pH 5.0 within 80 h), good cytocompatibility against NIH-3T3 cells and effective anticancer efficacy against MCF-7 cells. However, the star-shaped polymeric micelles exhibited preferable stability, which was confirmed by the lower critical micelle concentration (CMC 0.0034 mg/mL) and decrease rate of particle sizes after 7 days incubation (3.5%), compared with the linear polymeric micelle L-PLGA-D-P (CMC 0.0070 mg/mL, decrease rate of particle sizes was 9.6%). Overall, these developed polymeric micelles have promising application as drug delivery system in cancer therapy.

## 1. Introduction

Chemotherapy, in recent decades, has been considered as one of the most common therapy means for cancer in clinical practice and gained unprecedented attention [1]. However, the inherent flaws of anticancer drugs seriously affect the therapeutic efficacy of chemotherapy, mainly including low selectivity, probably poor bioavailability and untargeted distribution in the host body [2,3].

In an attempt to achieve improved therapeutic efficacy as well as minimal adverse effects, drug delivery systems with stimuli-responsive function that are able to release their drugs cargo in response to changes in pH, temperature or redox conditions have attracted more and more concerns [4,5,6,7]. Of these stimuli, pH-responsive amphiphilic polymers are currently of considerable academic and clinical interest since well-defined pH gradients exist in different tissues and cellular compartments, for instance, the pH of blood or normal tissues is 7.4, while the pH of extracellular environment and lysosomes of tumor tissues are about pH 6.4 and pH 5.0, respectively [8,9,10,11,12,13]. Xu and co-workers [14] developed a pH-responsive polymeric micelle based on block copolymer poly(ethylene glycol)-*b*-poly[2-(diisopropylamino) ethyl methacrylate], which exhibited proper stability in physiological environment and pH-triggered transforming capability between self-assembly and disassembly. Yang et al. [15] reported a pH-sensitive amphiphilic copolymer methyl poly(ethylene glycol) ether-*b*-poly(*β*-amino esters)-*b*-poly(lactic acid) and its self-assembled micelles, which showed good controlled release ability for doxorubicin (DOX) reflected by the obviously improved DOX release (96% of DOX) as pH decreased from pH 7.4 to pH 5.0. Qu et al. [16] prepared an amphiphilic pH-responsive micelle, poly[poly(ethylene glycol)methyl ether methacrylate-*b*-*N,N’*-di(methylamino)ethyl methacrylate-*b*-tert-butyl methacrylate], served as a platform for DOX delivery. This drug delivery system with pH-responsiveness displayed low leakage of DOX at pH 7.4 and rapid tunable drug release in intracellular environment with pH 5.0, reflected by 36% and 90% of DOX release at pH 7.4 and pH 5.0, respectively.

Despite the great process having achieved in pH-responsive polymeric micelles studies and application, there are still several considerable problems from the intravenous injection to anticancer drug release, such as insufficient stability [17,18,19], low drug loading capacity [6,20,21] and so on. Recently, in comparison to the reported polymers with small and soft aliphatic unit as core [22], the introduction of bulky and rigid building units as polymer core seems to be an effective strategy to improve the stability as well as drug loading capacity of the drug carriers [23,24,25,26]. Typically, Wang et al. [25,26] prepared a series of star-shaped polymeric micelles with rigid tetraphenylsilane core. These polymeric micelles showed relatively high stability and drug loading capacity, in contrast to that bearing soft aliphatic pentaerythritol core. In our previous work [23], two kinds of amphiphilic star-shaped polymers were synthesized based on adamantane and pentaerythritol core, respectively. It had been found that the polymeric micelle with bulky and rigid adamantane core exhibited higher thermodynamic stability and DOX loading capacity, reflected by the lower critical micelle concentration (CMC, 0.0050 mg/mL) and higher drug loading content (LC, 10.39%), compared to that with pentaerythritol core (CMC 0.0087 mg/mL, LC 8.94%).

**Figure 1:**
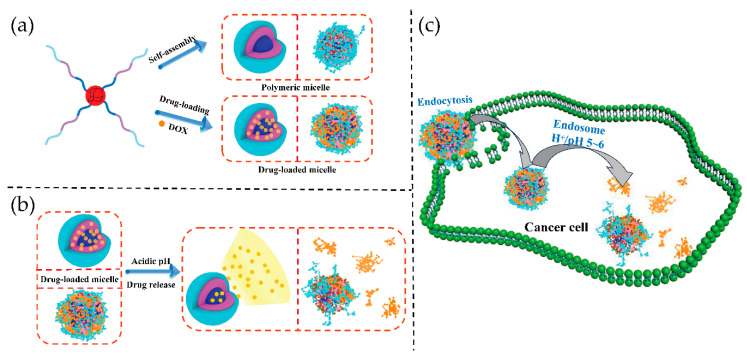
(**a**) Self-assembled behavior of star-shaped polymeric micelles and drug-loaded micelles. (**b**) Schematic illustration of controlled drug release triggered by pH for star-shaped drug-loaded micelles. (**c**) Internalization of DOX-loaded micelles by a cancer cell and the controlled release of DOX triggered by pH.

In our work, we focus on improving the stability and drug loading capacity of the polymeric micelles, as well as obtaining well-controlled release performance of anticancer drugs. Accordingly, linear (denoted as ‘L’) amphiphilic polymer, adamantane-poly(lactic-*co*-glycolic acid)-*b*-poly(*N,N’*-diethylaminoethyl methacrylate) poly(ethylene glycol) monomethyl ether (L-PLGA-D-P), and four arm star-shaped (denoted as ‘S’) amphiphilic polymer, adamantane-[poly(lactic-*co*-glycolic acid)-*b*-poly(*N,N’*-diethylaminoethyl methacrylate) poly(ethylene glycol) monomethyl ether]_4_ (S-PLGA-D-P), were designed and synthesized. Their self-assembled micelles were further developed to be platforms for anticancer drug delivery. To be specific, poly(lactic-*co*-glycolic acid) (PLGA) and poly(ethylene glycol) monomethyl ether (mPEG), as hydrophobic and hydrophilic segments, respectively, have the advantages of excellent biodegradability and compatibility [27]. Poly(*N,N’*-diethylaminoethyl methacrylate) (PDEAEMA) is hydrophobic in neutral or alkaline conditions, while become to be hydrophilic in acidic medium due to the protonation of amino group in PDEAEMA. Taking advantage of its interesting pH responsive behavior, thus, PDEAEMA is a suitable and promising pH responsive unit [28]. As one of the most effective anticancer drugs, DOX was widely used as the model drug to develop novel drug delivery system. Figure 1 illustrated the self-assembled behaviors of polymeric micelles, encapsulation of DOX, and controlled release of DOX in acidic microenvironment of cancer tissues. The properties of self-assembled polymeric micelles for anticancer drug delivery were extensively investigated, including CMC values, drug loading capacity, drug release behavior, in vitro cytotoxicity and anticancer efficacy. Furthermore, dissipative particle dynamics (DPD) simulation, as the auxiliary of the experimental results, was employed to understand the self-assembled behavior and dynamics DOX release process of DOX-loaded micelles.

## 2. Materials and Methods

### 2.1. Materials

1,4-Dioxan-2,5-dione (GA), D,L-lactide (D,L-LA) and copper (I) bromide (CuBr, 99%) were purchased from Sigma-Aldrich Co., Ltd. ( St. Louis, MO, USA). 1-Adamantanol (Ad-OH, 99%), stannous octoate (Sn(Oct)_2_, 95%), 2-bromoisobutyryl bromide (2-BIBB, 98%), 4-dimethylaminopyridine (DMAP), *N,N,N′,N′,N″*-pentamethyl diethylene-triamine (PMDETA, 98%), 2-(diethylamino) ethyl methacrylate (DEAEMA, 99%), doxorubicin hydrochloride (DOX·HCl) were purchased from Aladdin Bio-Chem Technology Co., Ltd. (Shanghai, China). Alkynyl-functionalized methoxypolyethylene glycols (alkynyl-mPEG, *M*_n_ = 2000) was purchased from Aichun Biological Technology CO., Ltd. (Shanghai, China). 1,3,5,7-Tetrahydroxyadamantane (Ad-(OH)_4_) was synthesized according to the reported literature [29]. Dulbecco’s Modified Eagle’s Medium (DMEM) and fetal bovine serum (FBS) were bought from the Biological Industries (BI, Beit-Haemek, Israel). Cell Counting Kit-8 (CCK-8) was obtained from Yeasen biotech Co., Ltd. (Shanghai, China). 96-Well plate was purchased from Sorfa Medical Co., Ltd. (Zhejiang, China). DEAEMA was purified via column-chromatography over neutral alumina to remove inhibitors before used. All other reagents were used without further purification unless stated otherwise.

### 2.2. Synthesis Methods

#### 2.2.1. Synthesis of Star-Shaped Macroinitiator Ad-[P(LA-*co*-GA)-Br]_4_

Ad-[P(LA-*co*-GA)-Br]_4_ was synthesized by the ring opening polymerization (ROP) and bromination reaction. Ad-(OH)_4_ (0.10 g, 0.5 mmol), D,L-LA (5.40 g, 37.5 mmol) and GA (1.45 g, 12.5 mmol) were placed in a flame-dried 100 mL Schlenk flask, then the system was degassed by 3 freeze-pump-thaw cycles. Subsequently, Sn(Oct)_2_ (68.5 mg, 1 wt% of D,L-LA and GA) was injected into the flask by syringe. The mixture was heated to 130 °C and stirred for 8 h. After cooling to room temperature, the crude product was dissolved in 50 mL dichloromethane (CH_2_Cl_2_), followed by adding dropwise to excess amount of cold *n*-hexane. The precipitated product was collected and then dried in a vacuum oven, resulting in white powdery Ad-[P(LA-*co*-GA)-OH]_4_. Next, Ad-[P(LA-*co*-GA)-OH]_4_ (*M*_n_, _GPC_ = 11657, 5.83 g, 0.5 mmol) dissolved in a mixture of anhydrous CH_2_Cl_2_ (50 mL) and triethylamine (TEA, 0.81 g, 8.0 mmol) was added to a flame-dried 100 mL Schlenk flask, which was degassed by 3 freeze-pump-thaw cycles. The system was cooled to 0 °C, and then 2-BIBB (1.84 g, 8.0 mmol) mixed with anhydrous CH_2_Cl_2_ was added slowly for a period of 2 h with vigorous stirring. The reaction was continued at room temperature for another 24 h. The mixture was washed successively with 1.0 mol/L HCl (100 mL), saturated NaHCO_3_ (100 mL), and water (100 mL). The organic solution was collected and dried with MgSO_4_ overnight followed by being removed excess solvent through rotary evaporation. After the concentrated solution was precipitated twice by excess amount of cold *n*-hexane, the product was collected and dried in a vacuum oven, resulting in yellowish powdery Ad-[P(LA-*co*-GA)-Br]_4_.

#### 2.2.2. Synthesis of Ad-[P(LA-*co*-GA)-*b*-PDEAEMA]_4_

Ad-[P(LA-*co*-GA)-*b*-PDEAEMA]_4_ was synthesized by atom transfer radical polymerizations (ATRP) of DEAEMA using Ad-[P(LA-*co*-GA)-Br]_4_ as macroinitiator. Typically, a flame-dried 100 mL Schlenk flask was charged with a mixture of Ad-[P(LA-*co*-GA)-Br]_4_ (5.99 g, 0.5 mmol), DEAEMA (5.56 g, 30.0 mmol), PMDETA (34.7 mg, 0.2 mmol) and anhydrous tetrahydrofuran (THF, 50 mL). Then, CuBr (29.3 mg, 0.2 mmol) was fed into the flask and the system was degassed by 3 freeze-pump-thaw cycles. After the polymerization was performed at 65 °C for 24 h, the system was cooled to room temperature and filled with air to terminate the chain growth. The mixture was diluted in THF (50 mL) and then passed through a neutral alumina column to remove the catalyst. After the organic solution was concentrated by rotary evaporation, the product was recovered by being precipitated into excess amount of cold *n*-hexane, and finally dried in a vacuum oven to obtain the yellowish solid product Ad-[P(LA-*co*-GA)-*b*-PDEAEMA]_4_.

#### 2.2.3. Synthesis of Star-Shaped Polymer S-PLGA-D-P

Polymer S-PLGA-D-P was prepared via Copper-Catalyzed Azide-Alkyne Cycloaddition (CuAAC) in ”click” chemistry. To be specific, Ad-[P(LA-*co*-GA)-*b*-PDEAEMA]_4_ (*M*_n, GPC_ = 19437, 3.89 g, 0.2 mmol) and NaN_3_ (130 mg, 2 mmol) dissolved in anhydrous *N,N’*-dimethylformamide (DMF, 30 mL) were transferred to a 100 mL round-bottom flask, and then the reaction was continued at 60 °C for 48 h. The mixture was cooled to room temperature, and precipitated twice by excess amount of cold deionized water. The precipitated Ad-[P(LA-*co*-GA)-*b*-PDEAEMA-N_3_]_4_ was collected by centrifugation and freeze-drying. To a flame-dried 100 mL Schlenk flask, alkynyl-mPEG (1.2 g, 0.6 mmol), Ad-[P(LA-*co*-GA)-*b*-PDEAEMA-N_3_]_4_ (1.94 g, 0.1 mmol), dipyridyl (9.4 mg, 0.06 mmol) and anhydrous DMF (50 mL) were added, followed by 3 freeze-pump-thaw cycles. Then, CuBr (8.6 mg, 0.06 mmol) was fed into the flask and the reaction was conducted at 60 °C for 48 h in the argon atmosphere. After cooling down to room temperature, the mixture was transferred to a dialysis bag (MwCO 7500) and dialyzed against deionized water for 72 h. The purified polymer was obtained by lyophilization.

#### 2.2.4. Synthesis of Linear Polymer L-PLGA-D-P

The linear polymer L-PLGA-D-P was synthesized according to the similar procedure to the S-PLGA-D-P described above, except that the used initiator was Ad-OH instead of Ad-OH_4_. The feed ratio of the materials and specific synthesis procedure were described, as shown in Section S1 (Supplementary Materials).

### 2.3. Preparation of the Blank Polymeric Micelles and DOX-Loaded Micelles

The blank polymeric micelles formed by polymers L-PLGA-D-P or S-PLGA-D-P were prepared according to the solvent evaporation method. Briefly, the prepared polymer (20 mg) was dissolved in 10 mL of acetone, and then added dropwise to 20 mL of deionized water under agitation. After the acetone was volatilized by continuously stirring at room temperature for 24 h, the solution was filtered through a 0.45 µm membrane filter to obtain the blank polymeric micelle solution. In addition, DOX-loaded micelles were fabricated by the diafiltration method [15,30]. DOX·HCl (20 mg or 50 mg) and TEA (0.04 mL or 0.10 mL) were dissolved in 10 mL of dimethyl sulfoxide (DMSO) and then stirred for 4 h to obtain a DOX base solution. After that, 100 mg of polymers were dissolved in 20 mL of DMSO at room temperature and then mixed with DOX base solution. After stirring overnight, the resulting solution was transferred to a dialysis bag (MwCO 3500) and dialyzed against deionized water for 48 h. The agglomerated polymers were removed by filtration with a 0.45 µm membrane filter. The dialysate was lyophilized and stored at −20 °C until use.

### 2.4. Characterization and Measurement

Nuclear magnetic resonance proton spectra (^1^H NMR) were recorded on a Bruker AVANVE III 400 MHz NMR spectrometer (Bruker, Billerica, MA, USA) for structural characterization of polymers. The ^1^H NMR spectra of polymers and CuAAC precursors were acquired using deuterated *N,N’*-dimethylformamide (DMF-*d*_7_) and deuterated chloroform (C*D*Cl_3_) as the deuterated solvents, respectively. Fourier transform infrared (FTIR) spectra were obtained using a Thermo Electron Nicolet-6700 FTIR spectrometer (Waltham, MA, USA) with KBr pellets, collected in the region of 4000–400 cm^−1^. The average molecular weight (*M*_n_) and dispersity (*M*_w_/*M*_n_) of polymers and their precursors were measured by gel permeation chromatographic (GPC) on a Waters 1515/2414 GPC equipment (Waters, Milford, MA, USA) equipped with polar gel columns. HPLC grade THF was used as the eluent with a flow rate of 1.0 mL/min and polystyrene (PS) was used as the standard of calibration for the column system. Dynamic light scattering (DLS, Brookhaven, New York, NY, USA) was used to measure the particle sizes of the blank polymeric micelles as well as DOX-loaded micelles with a concentration of 1.0 mg/mL, and the measurement angle was 90°. For the particle sizes of polymeric micelles under varying pH conditions, the phosphate buffer saline (PBS, pH 7.4 and 6.4) and acetate buffer (pH 5.0) were used as the incubation media. The characterization of DLS was repeated three times, and the results were obtained from three independent measurements. For collection of the Transmission electron microscopy (TEM) images, the polymeric micelles solution (1.0 mg/mL) was drop-cast on a carbon coated 200 mesh copper TEM grid, and after drying, these samples were then measured on a HT7700 apparatus (Hitachi, Japan) with an accelerating voltage of 120 kV.

### 2.5. CMC Measurement

Using pyrene as a fluorescence probe, the CMC values of L-PLGA-D-P and S-PLGA-D-P were determined by the fluorescence probe technique. Initially, pyrene was dissolved in acetone and the pyrene-acetone solution with a concentration of 6 × 10^−5^ M was obtained. Then, a series of polymer solutions were prepared at different concentrations ranging from 0.0001 to 0.1000 mg/mL. The polymer solutions were mixed with pyrene-acetone solution, and the concentration of pyrene was maintained as 6 × 10^−7^ M. Prior to measurement, the mixed solutions were equilibrated at room temperature in the dark for 24 h. The fluorescence intensity of all mixed solutions was recorded by a FluoroMax-4 fluorescence spectrometer (HORIBA Jobin Yvon, Clifton Park, NY, USA) with a bandwidth of 0.4 nm at room temperature. The emission wavelength was 373 nm and the fluorescence scanning range was from 300 nm to 350 nm.

### 2.6. Drug Loading and In Vitro Release

To determine the DOX loading capacity of polymeric micelles, 4.0 mg of DOX-loaded micelles were dissolved in 10 mL of DMSO, and then the UV absorption intensity of the solution was recorded at a wavelength of 480 nm by a Lambda 950 UV-Vis spectrophotometer (PerkinElmer, Waltham, MA, USA). Against the standard calibration curve of DOX (Figure S1, Supplementary Materials), DOX concentration of the above DOX-loaded micelles solution could be obtained. Furthermore, loading content (LC) and entrapment efficiency (EE) of DOX could be calculated according to the Equation (1) and Equation (2) [31]. All the experiments were repeated three times, and the results were obtained from three independent measurements.

(1) $$LC\;\left(\%\right)=\frac{\mathrm{Weight}\;\mathrm{of}\;\mathrm{DOX}\;\mathrm{loaded}}{\mathrm{Weight}\;\mathrm{of}\;\mathrm{DOX}\;\mathrm{loaded}\;\mathrm{micelles}}\times 100\%$$

(2) $$EE\;\left(\%\right)=\frac{\mathrm{Weight}\;\mathrm{of}\;\mathrm{DOX}\;\mathrm{loaded}}{\mathrm{Weight}\;\mathrm{of}\;\mathrm{DOX}\;\mathrm{in}\;\mathrm{feed}}\times 100\%$$

In vitro DOX release profiles of DOX-loaded micelles were measured in PBS (pH 7.4 and 6.4) or acetate buffer (pH 5.0). A total of 5.0 mg of lyophilized DOX-loaded micelles were dispersed in 5 mL of buffer solution, and then put into a dialysis bag (MwCO 3500). The dialysis bag was immersed in 40 mL of buffer solution with constant stirring (100 r/min) at 37 °C (body temperature). At each predefined time interval, 4 mL of buffer solution outside the dialysis bag was withdrawn and replaced by an equal volume of fresh media. The cumulative drug release amount (*E*_r_) was monitored by UV-Vis spectrophotometer and calculated based on the Equation (3). All the experiments were repeated three times, and the results were obtained from three independent measurements.

(3) $$E_{r}\;\left(\%\right)=\frac{V_{e}\sum_{1}^{n-1}C_{i}+V_{0}C_{n}}{m_{\mathrm{DOX}}}\times 100\%$$

where *V_e_* is the volume of buffer solution which was withdrawn (*V_e_* = 4 mL), *V_0_* is the total volume of the release medium (*V_0_* = 40 mL), *C_i_* (*C_n_*) is the concentration of DOX after *i* times (or *n* times) replacements of the buffer solution, *m*_DOX_ is the weight of DOX loaded in micelles.

### 2.7. In Vitro Cytotoxicity and Anticancer Efficacy Assay

In vitro cytotoxicity of polymeric micelles and DOX-loaded micelles were evaluated against NIH-3T3 cells by the standard CCK-8 assay. In detail, the NIH-3T3 cells were cultured onto a 96-well plate at a density of 3.0 × 10^3^ cells per well in complete DMEM culture medium containing 10% FBS and 1% P/S under 5% CO_2_ atmosphere at 37 °C for 24 h. Then, the growth medium was replaced with the complete DMEM culture medium containing the desired amounts of samples for 48 h incubation, while the fresh culture medium was utilized as the control group. After that, a 10 µL of CCK-8 was added to each well, and the cells were incubated for another 4 h. The absorbance of each well was measured at a wavelength of 450 nm by microplate reader (PerkinElmer, Waltham, MA, USA) to calculate the cell viability according to the Equation (4). Furthermore, In vitro anticancer efficacy of polymeric micelles and DOX-loaded micelles were carried out against MCF-7 cells with the similar method as described above, except that MCF-7 cells were cultured onto a 96-well plate at a density of 8.0 × 10^3^ cells per well. Typically, all the experiments were repeated three times, and the results were obtained from three independent measurements.

(4) $$\mathrm{Cell}\;\mathrm{viability}=\frac{A_{\mathrm{sample}}-A_{\mathrm{blank}}}{A_{\mathrm{control}}-A_{\mathrm{blank}}}\times 100\%$$

where *A*_sample_ is the absorbance of the wells treated with samples, *A*_control_ is the absorbance of the wells treated with control group, *A*_blank_ is the absorbance of the wells treated with neither cells nor samples.

### 2.8. Dissipative Particle Dynamics (DPD) Simulation

To understand the self-assembled behavior of polymeric micelles and DOX release process of DOX-loaded micelles, DPD simulation based on the coarse-grained models was performed by using the Materials Studio 8.0 (Accelrys Inc., San Diego, CA, USA), which was widely applied in other drug delivery systems [32,33,34]. The compositions of coarse grain models were shown in Figure S2. The elaborated DPD simulation methodology was described in Section S3 (Supplementary Materials) and the results of computational interaction parameters (*a*_ij_) were listed in Table S1.

## 3. Results and Discussion

### 3.1. Synthesis and Characterization of Polymers

**Scheme 1:**
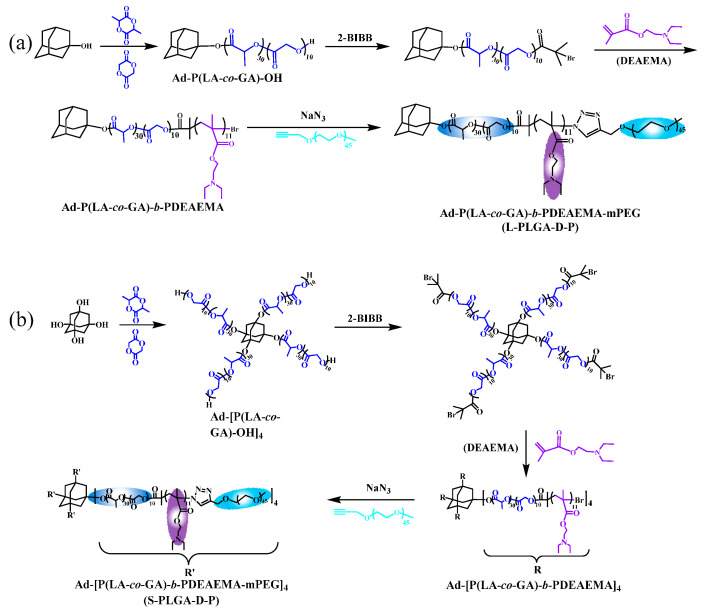
Synthetic routes of polymers L-PLGA-D-P (**a**) and S-PLGA-D-P (**b**).

The syntheses of polymers L-PLGA-D-P and S-PLGA-D-P were accomplished via a combination of ROP, ATRP and CuAAC methods using adamantane as a key starting material (Scheme 1). For polymer S-PLGA-D-P, Ad-(OH)_4_ was firstly used as an initiator to synthesize Ad-[P(LA-*co*-GA)-OH]_4_ by ROP method of D,L-LA and GA. Then, Ad-[P(LA-*co*-GA)-OH]_4_ was brominated with 2-BIBB to obtain Ad-[P(LA-*co*-GA)-Br]_4_. Using Ad-[P(LA-*co*-GA)-Br]_4_ as a macroinitiator, Ad-[P(LA-*co*-GA)-*b*-PDEAEMA]_4_ was synthesized through ATRP method of DEAEMA with a catalyst system of CuBr/PMDETA. Using Ad-[P(LA-*co*-GA)-*b*-PDEAEMA]_4_ and alkynyl-mPEG (*M*_n_ = 2000) as the precursors, polymer S-PLGA-D-P was obtained by CuAAC method with a CuBr/dipyridyl catalyst system. In addition, linear polymer L-PLGA-D-P was synthesized by ROP, ATRP and CuAAC methods using Ad-OH as the initiator, which was similar to the synthesis procedure of S-PLGA-D-P mentioned above.

**Figure 2:**
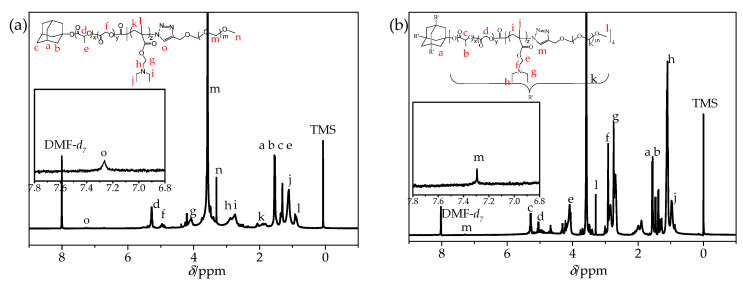
^1^H NMR spectra of L-PLGA-D-P (**a**) and S-PLGA-D-P (**b**).

The ^1^H NMR spectra of the prepared polymers and CuAAC precursors were depicted in Figure 2 and Figure S4, respectively. All of the peaks were well assigned to the corresponding protons. In Figure 2a, the signal at 1.55 ppm was ascribed to the resonance of the protons belonging to adamantane core, and the resonant signals at 1.55 ppm, 4.96 ppm and 5.28 ppm corresponded to –CH_3_, –CH_2_–, –CH– belonging to P(LA-*co*-GA) backbone, respectively. In addition, the signals of –CH_2_CH_2_– in PDEAEMA block adjacent to oxygen and nitrogen atoms were found at 2.88 ppm and 4.12 ppm, whereas the signals of –CH_2_– and –CH_3_ at the end of PDEAEMA block were found at 1.11 ppm and 2.76 ppm, respectively. The strong resonant signals at 3.58 ppm and 3.31 ppm corresponded to –CH_2_– and –CH_3_ in mPEG backbone, respectively. Importantly, the appearance of peak at 7.26 ppm corresponding to –CH– in the 1,4-disubstituted-1,2,3-triazole ring provided a direct evidence for the occurrence of CuAAC reaction. As for the ^1^H NMR spectrum of S-PLGA-D-P (Figure 2b), the signals for the corresponding protons were similar to those found in the ^1^H NMR spectrum of L-PLGA-D-P analyzed above, except that the adamantane core had one set of protons instead of three sets. ^1^H NMR (S-PLGA-D-P): *δ* 1.10 ppm (h), *δ* 1.55 ppm (a + b), *δ* 2.74 ppm (g), *δ* 2.92 ppm (f), *δ* 3.29 ppm (l), *δ* 3.58 ppm (k), *δ* 4.09 ppm (e), *δ* 4.99 ppm (d), *δ* 5.27 ppm (c), *δ* 7.30 ppm (m).

Furthermore, FTIR was used to track the terminal group variation of the CuAAC precursors and polymers. From the FTIR spectrum of Ad-P(LA-*co*-GA)-*b*-PDEAEMA-N_3_ shown in Figure S5a, after azidation of the Ad-P(LA-*co*-GA)-*b*-PDEAEMA, the typical absorption peak at 2112 cm^−1^ of the azido (–N_3_) can be observed, while the absorption peak at 565 cm^−1^ of the –Br end group in Ad-P(LA-*co*-GA)-*b*-PDEAEMA was disappeared. As for the FTIR spectrum of polymer L-PLGA-D-P, the absorption peaks of the –N_3_ in Ad-P(LA-*co*-GA)-*b*-PDEAEMA-N_3_ and –C≡C–H (2166 cm^−1^) in alkyne mPEG were shown to disappear due to the subsequent CuAAC reaction [35,36]. Following a similar analysis, the terminal group variation of the S-PLGA-D-P and its CuAAC precursors were also confirmed of which characterized results were provided in Figure S5b.

The average molecular weights (*M*_n_) and distributions of the polymers were further determined by GPC analyses, as shown in Figure S6 and Table 1. GPC curves (Figure S6) of the polymers and their precursors exhibited monomodal symmetric distributions, indicating a well-controlled process of the ATRP polymerizations for DEAEMA and the CuAAC reaction. Meanwhile, the GPC curves of L-PLGA-D-P and S-PLGA-D-P apparently shifted toward left relative to that of their precursor indicative of the increase of molecular weights, which were conformed to the theoretical values. Moreover, the resultant polymers L-PLGA-D-P and S-PLGA-D-P displayed narrow molecular weight distribution (*M*_w_/*M*_n_ < 1.50), which are available for further application in anticancer drugs delivery [8].

### 3.2. Self-Assembly and Stability of Amphiphilic Polymeric Micelles

The amphiphilic nature endows the resultant polymers with self-assembled ability to form core-shell micelles. Figure S7 (Supplementary Materials), presenting the ^1^H NMR spectra of L-PLGA-D-P (a) and S-PLGA-D-P (b) in deuterated water (D_2_O), showed the -CH_2_- characteristics peak of hydrophilic mPEG segment at 3.58 ppm, whereas the signals corresponding to hydrophobic P(LA-*co*-GA) and PDEAEMA segments were absent, indicative of the micellar structure with hydrophobic P(LA-*co*-GA) and PDEAEMA internal core surrounded by a shell composed with hydrophilic mPEG.

**Figure 3:**
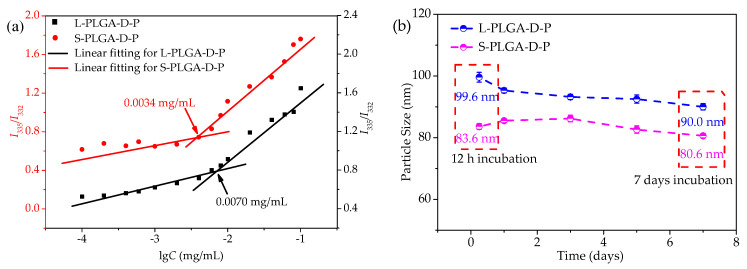
(**a**) Dependency of the ratios of fluorescent intensity (*I*_335_/*I*_332_) on polymer concentration in aqueous solution. (**b**) The effects of incubate time on the particle sizes of polymeric micelles in PBS with pH 7.4 at room temperature determined by DLS.

The stability is one of the key parameters of drug delivery system, and it is dependent on the Gibbs free energy of system that is approximately proportional to the CMC values. Typically, the lower CMC value means a more negative Gibbs free energy, reflecting the higher stability of drug delivery system [37]. In our work, the CMC values of polymers L-PLGA-D-P and S-PLGA-D-P were measured in aqueous solutions by fluorescence spectroscopy using pyrene as a fluorescence probe. The ratios of fluorescent intensity *I*_335_/*I*_332_ were plotted against polymer concentrations, and the intersection of two fitting lines was corresponded to the CMC values [22], as shown in Figure 3a. It could be observed that polymer S-PLGA-D-P displayed quite low CMC value with 0.0034 mg/mL reflecting that its self-assembled polymeric micelles owned desired stability, and its CMC value could be comparable to that of many reported pH-responsive polymers shown in Table 2. Interestingly, it could be found that, with the nearly identical block ratio for one arm, polymer S-PLGA-D-P displayed a lower CMC value than L-PLGA-D-P (CMC 0.0070 mg/mL), demonstrating that the polymeric micelle with star-shaped topology had considerably higher stability in contrast to the linear counterpart. In addition, the stability of polymeric micelles was further characterized by monitoring their size variation during long-term incubation for 7 days in PBS. Figure 3b displayed that the particle sizes of L-PLGA-D-P and S-PLGA-D-P were decreased from 99.6 nm and 83.6 nm to 90.0 nm and 80.6 nm, respectively, after 7 days incubation. The decrease rates were 9.6% for L-PLGA-D-P and 3.5% for S-PLGA-D-P, which indicated that S-PLGA-D-P had higher stability rather than L-PLGA-D-P. Thus, based on the analysis above, star-shaped polymeric micelle S-PLGA-D-P owned a better stability which was crucial and advantages to maintain the integrity of the polymeric micelles upon large-ratio blood dilution post intravenous dose administration [17].

### 3.3. pH Response of Polymeric Micelles

**Figure 4:**
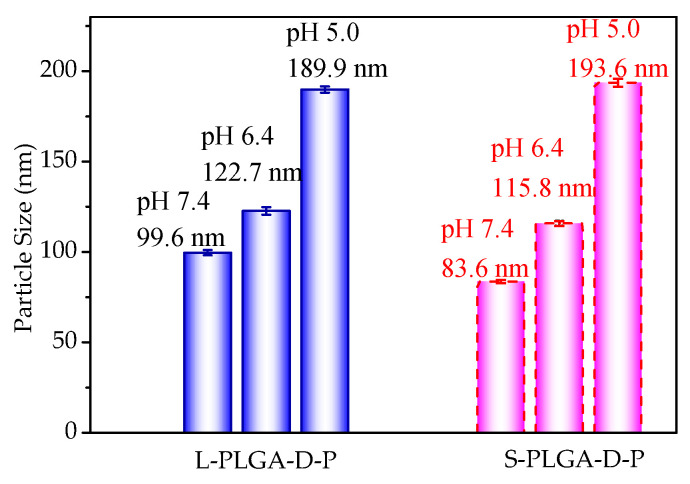
The size variations of polymeric micelles with pH values.

**Figure 5:**
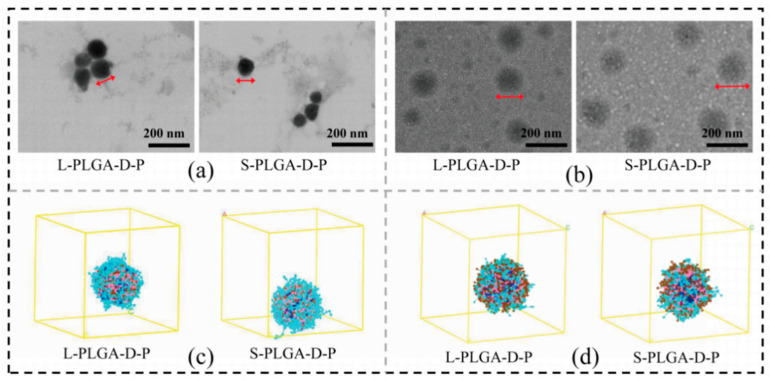
TEM images of self-assembled polymeric micelles at pH 7.4 (**a**) and pH 5.0 (**b**). DPD equilibrium states of polymeric micelles at neutral (**c**) and acidic condition (**d**). The beads of Ad, LA, GA, MAA, DEA (DEAH), TriAZO and mPEG were represented in red, blue, indigo, pink, brown, purple and light blue, respectively.

The pH-responsive function of polymeric micelles was investigated by means of DLS and TEM. Figure 4 showed the particle sizes (*D*_h_) of polymeric micelles under varying pH conditions (pH 7.4, 6.4 and 5.0) after 12h incubation, and the Figure S8 (Supplementary Materials) presented the corresponding particle size distribution. At pH 7.4, the *D*_h_ of L-PLGA-D-P and S-PLGA-D-P were 99.6 nm and 83.6 nm, respectively, which remarkably grew to be 189.9 nm and 193.6 nm with the reduction of pH values from 7.4 to 5.0. This pronounced phenomenon could be attributed to the conversion of PDEAEMA from hydrophobicity to hydrophilicity at a varying pH value. Under neutral condition, PDEAEMA segment was relatively hydrophobic leading to the compact polymeric micelles. Under the acidic condition, however, the pendant tertiary amine groups in PDEAEMA were protonated and become to be hydrophilic, leading to the loose and turgid polymeric micelles [38]. Moreover, the pH-responsive function of polymeric micelles was further supported by TEM observation. From Figure 5a,b, it could be observed that the polymeric micelles showed spherical morphology. At pH 5.0, the sizes of polymeric micelles became apparently larger than those observed at pH 7.4, which was in accordance with the variation trend of *D*_h_ obtained by DLS. Consequently, the results of DLS and TEM indicated that both the linear and star-shaped polymeric micelles had desired pH-responsive behavior. In addition, with the decrease of pH, the transition of pendant tertiary amine groups in PDEAEMA segment from hydrophobicity to hydrophilicity was further understood through DPD simulation. For the equilibrium states of L-PLGA-D-P and S-PLGA-D-P at neutral condition, as seen in Figure 5c, the components finally formed a large single spherical micelle which was in quite good agreement with that observed in TEM images. The hydrophobic P(LA-*co*-GA) and PDEAEMA segments self-assembled to form the inner core surrounded by the mPEG shell (light blue), which was consistent with the self-assembled behavior of the resultant polymers stated in Section 3.2 [33]. In contrast, at acidic condition, it could be observed that the protonated DEA groups (DEAH beads) were exposed to the surface of spherical micelle because the PDEAEMA became to be hydrophilic with the decrease of pH.

### 3.4. Properties of DOX-Loaded Micelles

The DOX-loaded micelles were prepared by the classical dialysis protocol with the feed ratio of DOX/polymer of 20/100 mg/mg or 50/100 mg/mg. Their particle sizes were detected using DLS, while the DOX loading capacity reflected by drug loading content (LC) and encapsulation efficiency (EE) were detected using UV-Vis. From Table 2, DOX-loaded micelles displayed the larger particle sizes than their corresponding blank polymeric micelles, and the larger particle sizes were obtained with more DOX fed to the polymeric micelles. Notably, the particle sizes of DOX-loaded micelles were less than 200 nm, which was reportedly beneficial to the perfusion and accumulation of drug-loaded micelles in tumor tissues by enhanced permeation and retention (EPR) mechanism [39,40,41]. As the feed ratio of DOX/polymer increased, the LCs of DOX-loaded micelles accordingly increased, reflecting that the feed ratio of DOX/polymer had an important impact on LCs. It is noteworthy that, in particular, the LCs of DOX@S-PLGA-D-P were up to 12.4% and 22.9% with the feed ratio of DOX/polymer of 20/100 mg/mg and 50/100 mg/mg, respectively, which were comparable to that of the reported polymeric micelles (Table 2), such as 4sPCLDEAS (LC = 11.4%, DOX/polymer of 20/100 mg/mg) [42], (PCL)_3_-(PDEAEMA-*b*-PPEGMA)_3_ (LC = 12.8−19.6%, DOX/polymer of 50/100 mg/mg) [8]. The desired drug-loading capacity of polymeric micelles might be attributed to the bulky and rigid adamantane core as well as hydrophobic PLGA and PDEAEMA segments, which were advantageous to entrap more drugs. In our previous works [10,23,38], it was manifested that the length of hydrophobic segments played an important role in drug loading capacity of drug-loaded micelles, for instance, longer hydrophobic segments resulted in higher LCs values. Interestingly, with the nearly identical blocks ratio of polymer for one arm, the LCs of DOX@S-PLGA-D-P and DOX@L-PLGA-D-P were 22.9% and 21.6%, respectively, which demonstrated that the topological structure of star-shaped and linear polymers did not seem to be a key factor for drug loading capacity of the DOX-loaded micelles.

Maintaining the same fraction of DOX/polymer (50/100 mg/mg) as the experiment, the aggregation morphologies and cross-section views of DOX-loaded micelles obtained by DPD simulation were shown in Figure S3 (Supplementary Materials). It could be found that the formation process of DOX-loaded micelles which was finally formed a large single spherical micelle was similar to that of the blank polymeric micelles (Figure 5c). The cross-section views of DOX-loaded micelles showed that the DOX was mainly distributed in the middle layer (PDEAEMA segment) of polymeric micelles structure. This phenomenon could be attributed to the greater affinity between DOX and PDEAEMA than that between DOX and P(LA-*co*-GA), reflected by the lower interaction parameters of DOX and PDEAEMA (Table S1, Supplementary Materials). Furthermore, DOX was diffused to P(LA-*co*-GA) inner core which required to overcome the interspace resistance of the PDEAEMA middle layer [46]. Combining with the DPD equilibrium state and cross-section views of these DOX-loaded polymeric micelles, it could be found that they have the similar self-assembled behavior, thought they had different topology. Besides, the length of PDEAEMA segment for the arms of L-PLGA-D-P and S-PLGA-D-P were nearly identical, and it could be observed that the cross-section views of DOX-loaded micelles (Figure S3) displayed the nearly identical thickness of PDEAEMA middle layer in which the DOX was mainly distributed. Therefore, it could seem to support the experimental conclusion that polymeric micelles L-PLGA-D-P and S-PLGA-D-P showed the comparable DOX loading capacity.

### 3.5. In Vitro Drug Release Behavior

**Figure 6:**
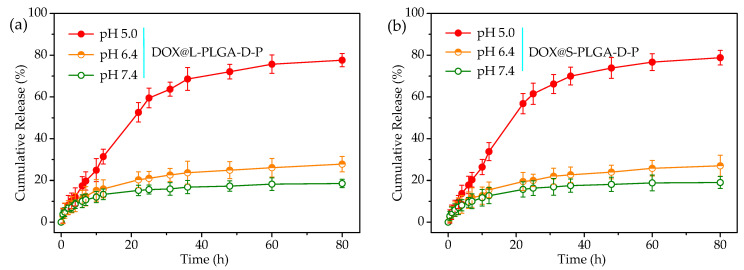
In vitro drug release curves of DOX@L-PLGA-D-P (**a**) and DOX@S-PLGA-D-P (**b**) under different pH conditions (pH 7.4, 6.4 and 5.0).

*In vitro* drug release behavior of DOX-loaded micelles with the feed ratio of DOX/polymer of 50/100 mg/mg was studied under different pH condition (pH 7.4, 6.4 and 5.0) at 37 °C aimed to mimic the microenvironment of normal tissues and cancer tissues. As seen in Figure 6, at pH 7.4, only 13.3% and 12.7% of DOX were released from DOX@L-PLGA-D-P and DOX@S-PLGA-D-P within 12 h, respectively, while the release rate increased tardily and tended toward stability within 80 h. It meant that the DOX-loaded micelles could remain stable and provide a good protection for DOX avoiding the excess leakage of DOX in the blood circulation of normal physiological condition. In addition, in the simulated external microenvironment of cancer tissues (pH 6.4), the DOX cumulative release of DOX@L-PLGA-D-P and DOX@S-PLGA-D-P were slightly accelerated and reached to 27.8% and 27.0% within 80 h, respectively. Conversely, as expected, the release rate of DOX obviously expedited when the pH dropped from 7.4 to 5.0, and the DOX cumulative release of DOX@L-PLGA-D-P and DOX@S-PLGA-D-P could reach to 77.6% and 78.8% at pH 5.0 after 80 h, respectively. It could be indicative of the evident pH-controlled drug release behavior of DOX-loaded micelles which profited from their strong pH-responsiveness. Such interesting pH-controlled drug release behavior could be ascribed to the protonation of tertiary amine groups in PDEAEMA which became to be hydrophilic under an acidic condition of tumor tissues [44,47]. In addition, similar DOX release behavior of DOX@L-PLGA-D-P and DOX@S-PLGA-D-P could be observed from the in vitro drug release profiles. This could be ascribed to the similar self-assembled behavior of DOX-loaded micelles and the almost identical length of DEAEMA segment for these two polymers, which play a key role in the DOX releasing properties [25].

**Figure 7:**
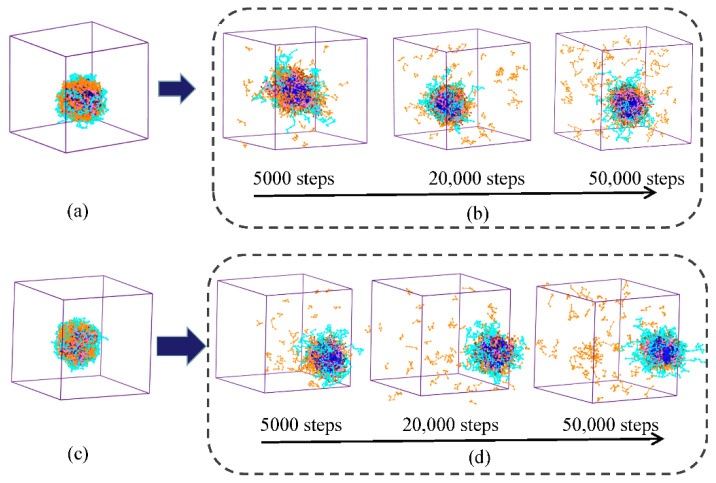
(**a**) DPD equilibrium state of DOX@L-PLGA-D-P under neutral condition. (**b**) DPD morphologies of DOX@L-PLGA-D-P at different simulation time under acidic condition. (**c**) DPD equilibrium state of DOX@S-PLGA-D-P under neutral condition. (**d**) DPD morphologies of DOX@S-PLGA-D-P at different simulation time under acidic condition. The beads of Ad, LA, GA, MAA, DEA (DEAH), TriAZO, mPEG and DOX were represented in red, blue, indigo, pink, brown, purple, light blue and orange, respectively.

The dynamics release process of DOX in the acidic aqueous solution was further understood at mesoscopic scale taking advantage of DPD simulation. As shown in Figure 7a, under neutral condition, DOX@L-PLGA-D-P was formed to be a spherical drug-loaded micelle in DPD equilibrium state. However, from Figure 7b, it could be found that DOX was released from DOX-loaded micelles under acidic condition and more DOX was released with the increase of DPD simulation time. In addition, the dynamics release process of DOX@ S-PLGA-D-P was similar to that of DOX@L-PLGA-D-P, which was in line with the results that DOX@ L-PLGA-D-P and DOX@ S-PLGA-D-P had similar DOX release behavior discussed above. In the equilibrium state of drug-loaded micelles under acidic condition, although most of DOX was release, a small amount of DOX enclosed within the inner core of DOX-loaded micelles failed to be effectively released. This phenomenon agreed with the fact that the in vitro DOX cumulative release of DOX-loaded micelles did not reach 100% within 80 h.

### 3.6. In Vitro Cytotoxicity

**Figure 8:**
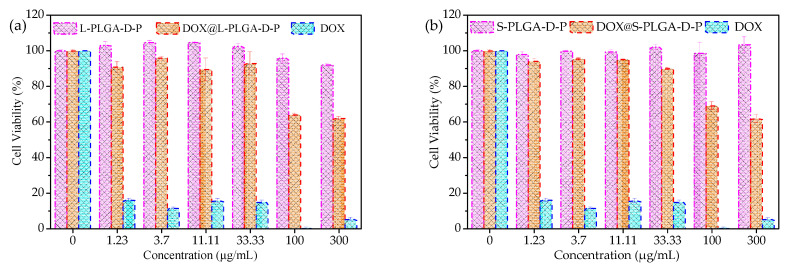
Cytotoxicity assay cultured with (**a**) L-PLGA-D-P, DOX@L-PLGA-D-P, free DOX and (**b**) S-PLGA-D-P, DOX@S-PLGA-D-P, free DOX for 48 h incubation at different concentrations of samples against NIH-3T3 cells.

The cytocompatibility of polymeric micelles and DOX-loaded micelles against NIH-3T3 cells were evaluated in vitro through CCK-8 assay. Figure 8 depicted the dose-dependent cell viability of NIH-3T3 cells cultured with L-PLGA-D-P (a), S-PLGA-D-P (b) and their corresponding DOX-loaded micelles. The polymeric micelles L-PLGA-D-P and S-PLGA-D-P showed negligible cytotoxicity to NIH-3T3 cells in the ranging of 0–300 µg/mL reflected by the high viability of NIH-3T3 cells (more than 95%), demonstrating their good cytocompatibility. This could be mainly attributed to the fact that all the segments owned the good biocompatibility in the polymer composition. Typically, a high cytotoxicity was induced by free DOX for NIH-3T3 cells, even at low dose (1.23 µg/mL), which manifested the toxic side effects of anticancer drug DOX on normal tissues. Satisfactorily, as for DOX-loaded micelles, no apparent cytotoxicity was observed when the concentration of DOX-loaded micelles was lower than 33.33 µg/mL. However, the increased cytotoxicity was detected with the higher concentration of DOX-loaded micelles. This phenomenon was understandable which was ascribed to that DOX-loaded micelles showed a relatively low amount of DOX cumulative release under normal physiological condition (pH 7.4) for 48 h incubation (17.3% for DOX@L-PLGA-D-P and 18.1% for DOX@S-PLGA-D-P), and thus, the higher concentration of DOX-loaded micelles resulted in the higher additional cumulative release of DOX which was highly toxic to NIH-3T3 cells. Moreover, from Table S2 (Supplementary Materials), no half maximal inhibitory concentration (IC_50_) was observed at the tested concentration, which was another powerful demonstration of good cytocompatibility for polymeric micelles and DOX-loaded micelles.

### 3.7. In Vitro Anticancer Efficacy

**Figure 9:**
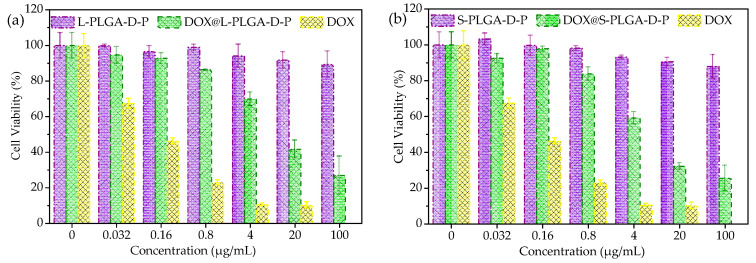
Viability of MCF-7 cells cultured with (**a**) L-PLGA-D-P, DOX@L-PLGA-D-P, free DOX and (**b**) S-PLGA-D-P, DOX@S-PLGA-D-P, free DOX for 48 h incubation.

The in vitro anticancer efficacy of polymeric micelles and DOX-loaded micelles against MCF-7 cells were carried out by the CCK-8 assay. Figure 9 showed the dose-dependent cell viability of MCF-7 cells cultured with L-PLGA-D-P (a), S-PLGA-D-P (b) and their corresponding DOX-loaded micelles. It could be found that a fairly high viability of MCF-7 cells, approximately 90%, was still maintained after 48 h incubation at concentrations of blank polymeric micelles up to 100 µg/mL, indicating that the obtained polymeric micelles do not exhibit effective anticancer efficacy on MCF-7 cells. For free DOX, it showed remarked therapeutic efficacy against MCF-7 cells, as the viability of MCF-7 cells was significantly declining with the increasing concentration of DOX. More importantly, the in vitro viability of MCF-7 cells culturing with DOX@L-PLGA-D-P and DOX@S-PLGA-D-P decreased from 92.5% to 32.0% and from 94.6% to 41.6%, respectively, as the concentrations of samples increased from 0.032 to 20 µg/mL, indicative of the effective therapeutic efficacy of DOX-loaded micelles. Interestingly, the viability of MCF-7 cells culturing with DOX-loaded micelles was quite different from that of NIH-3T3 cells, which was in line with the in vitro drug release profiles with well-controlled release behavior. The phenomenon could be ascribed to the different DOX release behavior of DOX-loaded micelles in the microenvironment of NIH-3T3 cells and MCF-7 cells. In detail, in the microenvironment of NIH-3T3 cells (pH 7.4), DOX-loaded micelles provided a good protection for DOX which could avoid the excess leakage of DOX, and the cell viability of NIH-3T3 cells was relatively high indicating the good cytocompatibility of DOX-loaded micelles. In the microenvironment of MCF-7 cells (pH 5.0), however, DOX-loaded micelles displayed the accelerated release of DOX because of their strong pH-responsiveness, resulting in the effective therapeutic efficacy of DOX-loaded micelles, and therefore, the viability of MCF-7 cells was shown to decrease as the concentration of DOX-loaded micelles increased. By combining with the low IC_50_ values of DOX-loaded micelles against MCF-7 cells that 14.600 µg/mL for DOX@L-PLGA-D-P and 9.535 µg/mL for DOX@S-PLGA-D-P (Table S2, Supplementary Materials), the results indicated an obvious anticancer efficacy of DOX-loaded micelles.

## 4. Conclusions

In summary, two types of polymers with linear (L-PLGA-D-P) and star-shaped (S-PLGA-D-P) topology were successfully synthesized by ROP, ATRP and CuAAC methods using adamantane as a key starting material. Their self-assembled polymeric micelles were prepared and applied for a promising anticancer drug carrier using DOX as the model drug. These two types of polymeric micelles showed good drug loading capacity up to be 21.6% and 22.9% for L-PLGA-D-P and S-PLGA-D-P, respectively, which were comparable to that of the reported polymeric micelles. In vitro DOX release profiles showed the prepared DOX-loaded micelles displayed desired pH-responsive and controlled release behavior as reflected by the less leakage of DOX in normal physiological condition with pH 7.4 as well as accelerated DOX release behavior at pH 5.0. Furthermore, the in vitro cell assays demonstrated that the polymeric micelles and DOX-loaded micelles had good biocompatibility against normal tissues, and meanwhile, DOX-loaded micelles possessed effective inhibition for proliferation of MCF-7 cells. Nevertheless, with the almost identical ratio of the blocks of the polymers, the star-shaped polymeric micelle S-PLGA-D-P exhibited merit of enhanced micelle stability reflected by its lower CMC value (0.0034 mg/mL) and decrease rate of particle sizes after 7 days incubation (3.5%) compared with linear polymeric micelle L-PLGA-D-P (CMC 0.0070 mg/mL, decrease rate of particle sizes was 9.6%), which manifested that the star-shaped topology was beneficial to improve the stability of polymeric micelles compared with the linear topology. In consideration of the properties described above, polymeric micelles L-PLGA-D-P and S-PLGA-D-P showed good potential in the application field of anticancer drug delivery with well-controlled release for safe and highly efficient cancer therapy.

## Figures and Tables

**Table 1:** Molecular weights and distributions of polymers.

Samples	^a^ *M*_n,th_	*M*_n,GPC_	*M*_w_/*M*_n_
Ad-P(LA-*co*-GA)-*b*-PDEAEMA	5834	5110	1.36
L-PLGA-D-P	7834	7077	1.28
Ad-[P(LA-*co*-GA)-*b*-PDEAEMA]_4_	22,772	19,437	1.37
S-PLGA-D-P	30,772	27,002	1.41

^a^ Calculated by theoretical analysis from the feed ratio of monomers to initiator.

**Table 2:** Loading content, encapsulation efficiency and particle sizes of DOX-loaded micelles in our work and the reported micelles.

Samples	DOX/Polymer (mg/mg)	LC (%)	EE (%)	^a^*D*_h_ (nm)	^b^ CMC (mg/mL)
DOX@L-PLGA-D-P	0/10	—	—	99.6 (±2.1)	0.0034
	20/100	12.1 (±0.3)	57.6 (±0.5)	126.3 (±1.6)	
	50/100	21.6 (±0.6)	49.2 (±0.6)	153.4 (±3.2)	
DOX@S-PLGA-D-P	0/10	—	—	83.6 (±3.0)	0.0070
	20/100	12.4 (±0.2)	58.2 (±0.8)	120.6 (±2.0)	
	50/100	22.9 (±0.5)	53.3 (±1.1)	156.6 (±2.6)	
PT-(PLGA-SS-mPEG)_4_ [23]	20/100	6.66	39.7	132	0.0087
(PCL)-(PDEAEMA-*b*-PPEGMA)_3_ [8]	50/100	12.8–19.6	29.4–48.8	167–232	0.0035
4sPCLDEAS [42]	20/100	11.4	51.5	113.7	—
6AS-PCL-PAA-PPEGMA [43]	14/100	12.0	—	—	—
*β*-CD-(PLA-PDMAEMA-PEtOxMA)_21_ [44]	25/100	13.7	61.0	—	—
	50/100	21.7	59.1	—	
4AS-PLC-*b*-PDMAEMA [45]	50/100	8.64	37.8	102.7	0.0041

^a^ Particle sizes determined by DLS. ^b^ CMC values of the corresponding polymers which could self-assembly into DOX-loaded micelles with DOX.

## Data Availability

The date are available upon request from the corresponding author.

## Supplementary Materials

The following are available online at https://www.mdpi.com/2079-4991/11/1/188/s1, Figure S1: Standard calibration curve of DOX in DMSO, Figure S2: Coarse grain models of (a) L-PLGA-D-P, (b) L-PLGA-DH-P, (c) S-PLGA-D-P, (d) S-PLGA-DH-P, (e) DOX, (f) DOXH, (g) Water, Figure S3: DPD equilibrium states of DOX-loaded L-PLGA-D-P micelles (a) and DOX-loaded S-PLGA-D-P micelles (b). Cross-section views of DOX-loaded L-PLGA-D-P micelles (c) and DOX-loaded S-PLGA-D-P micelles (d), Figure S4: ^1^H NMR spectra of polymers L-PLGA-D-P (a), S-PLGA-D-P (b) and the CuAAC precursors, Figure S5: FTIR spectra of polymers L-PLGA-D-P (a), S-PLGA-D-P (b) and their CuAAC precursors, Figure S6: GPC traces of L-PLGA-D-P, S-PLGA-D-P and their precursors, Figure S7: ^1^H NMR spectra of L-PLGA-D-P (a) and S-PLGA-D-P (b) in D_2_O, Figure S8: Particle size distribution of L-PLGA-D-P determined by DLS after 12 h incubation, (a) pH 7.4, (b) pH 6.4, (c) pH 5.0. Particle size distribution of S-PLGA-D-P determined by DLS after 12 h incubation, (d) pH 7.4, (e) pH 6.4, (f) pH 5.0, Table S1: Interaction parameters *a_ij_* between different beads used in DPD simulation, Table S2: IC_50_ values of blank micelles, DOX-loaded micelles and free DOX.
