# Supplementary material for: Acid-Responsive Adamantane-Cored Amphiphilic Block Polymers as Platforms for Drug Delivery
Source: Nanomaterials (Basel). 2021 Jan 13;11(1):188. doi: 10.3390/nano11010188 (PMC7828523; doi:10.3390/nano11010188)
Supplement: Supplementary file 1 [file nanomaterials-11-00188-s001.pdf]

Supplementary Materials: Acid-Responsive Adamantane-Cored Amphiphilic Block Polymers as Platforms for Drug Delivery

Weiqiu Wen [1], Chong Guo [2] and Jianwei Guo [1,*]

Table of contents

S1 Detailed Synthesis Procedure.. 1

S2 Standard Calibration Curve of DOX in DMSO.. 2

S3 Dissipative Particle Dynamics (DPD) Simulation Methodology.. 3

S4 ^{1}H NMR Spectra of as-Synthesized Polymers and their Precursors 5

S5 FTIR Spectra of as-Synthesized Polymers and their Precursors... 6

S6 GPC Results of the Polymers and their Precursors 7

S7 ^{1}H NMR Spectra of Polymers in Deuterated Water (D_2O).. 8

S8 Particle Size Distribution of Polymeric Micelles Determined by DLS with Different pH Values. 9

S9 IC_{50} Values of Polymeric Micelles, DOX-loaded Micelles and Free DOX............................... 10

S1 Detailed Synthesis Procedure

S1.1 Synthesis of Linear Macroinitiator Ad-P(LA-co-GA)-Br

Ad-OH (0.33 g, 2.0 mmol), D,L-LA (5.40 g, 37.5 mmol) and GA (1.45 g, 12.5 mmol) were placed in a flame-dried 100 mL Schelenk flask and then the system was degassed by 3 freeze-pump-thaw cycles. Subsequently, $Sn(Oct)_2$ (68.5 mg, 1 wt% of the total amount of D,L-LA and GA) was injected into the flask by syringe. The mixture was heated to 130 °C and stirred for 8h. After cooling to room temperature, the crude product was dissolved in 50 mL dichloromethane (CH_2Cl_2), followed by adding dropwise to excess amount of cold *n*-hexane to precipitate the product which was collected and dried under vacuum for 24 h, resulting in white powdery Ad-P(LA-*co*-GA)-OH. Next, Ad-P(LA-*co*-GA)-OH ($M_{n,\,GPC}$=3057, 3.06 g, 1.0 mmol), dissolved in a mixture of anhydrous CH_2Cl_2 (50 mL) and triethylamine (TEA, 0.40 g, 4.0 mmol), was added to a flame-dried 100 mL Schelenk flask and the system was degassed by 3 freeze-pump-thaw cycles. The system was cooled to 0 °C and then the 2-BIBB (0.92 g, 4.0 mmol) mixed with anhydrous CH_2Cl_2 was added slowly for a period of 2 h with vigorous stirring. The reaction was continued at room temperature

for another 24 h. The mixture was washed successively with 1 mol/L HCl (100 mL), saturated $NaHCO_3$ (100 mL), and water (100 mL). The organic solution was collected and dried with $MgSO_4$ overnight followed by rotary evaporation to remove excess solvent. After the concentrated solution was precipitated twice by excess amount of cold *n*-hexane, the product was collected and dried under vacuum for 24 h, resulting in yellowish powdery Ad-P(LA-*co*-GA)-Br.

S1.2 Synthesis of Ad-P(LA-co-GA)-b-PDEAEMA

A flame-dried 100 mL Schlenk flask was charged with a mixture of Ad-P(LA-*co*-GA)-Br (3.14 g, 1.0 mmol), DEAEMA (2.78 g, 15.0 mmol) and PMDETA (17.3 mg, 0.1 mmol) and anhydrous tetrahydrofuran (THF, 50 mL). Then, a required amount of CuBr (14.7 mg, 0.1 mmol) was fed into the flask and the system was degassed by 3 freeze-pump-thaw cycles. After the polymerization was performed at 65 °C for 24 h, the system was cooled to room temperature and filled with air to terminate the chain growth. The mixture was diluted in THF (50 mL) and then passed through a neutral alumina column to remove the catalyst. After the organic solution was concentrated by rotary evaporation, the product was recovered by being precipitated into excess amount of cold *n*-hexane and finally dried under vacuum for 24 h to obtain the yellowish solid product Ad-P(LA-*co*-GA)-*b*-PDEAEMA.

S1.3 Synthesis of Linear Polymer L-PLGA-D-P

Ad-P(LA-*co*-GA)-*b*-PDEAEMA ($M_{n, GPC}$ = 5110, 1.02 g, 0.2 mmol) and NaN_3 (130 mg, 2 mmol) dissolved in anhydrous *N,N'*-dimethylformamide (DMF, 30mL) were transferred to a 100 mL round-bottom flask, and the reaction was continued at 60 °C for 48 h. The mixture was cooled to room temperature, and precipitated twice by excess amount of cold deionized water. The precipitated Ad-P(LA-*co*-GA)-*b*-PDEAEMA-N_3 was collected by centrifugation and freeze-drying. To a flame-dried 100 mL Schlenk flask, subsequently, alkyne-mPEG (1.2 g, 0.6 mmol), Ad-P(LA-*co*-GA)-*b*-PDEAEMA-N_3 (0.51 g, 0.4 mmol) and dipyridyl (9.4 mg, 0.06 mmol) were added and dissolved in 50 mL anhydrous DMF, followed by 3 freeze-pump-thaw cycles. Then, CuBr (8.6 mg, 0.06 mmol) was fed into the flask and the reaction was conducted at 60 °C for 48 h in the argon atmosphere. After cooling down to the room temperature, the mixture was transferred to a dialysis bag (MwCO 3500) and dialyzed against deionized water for 72 h. The purified polymer L-PLGA-D-P was obtained by lyophilization.

S2 Standard Calibration Curve of DOX in DMSO

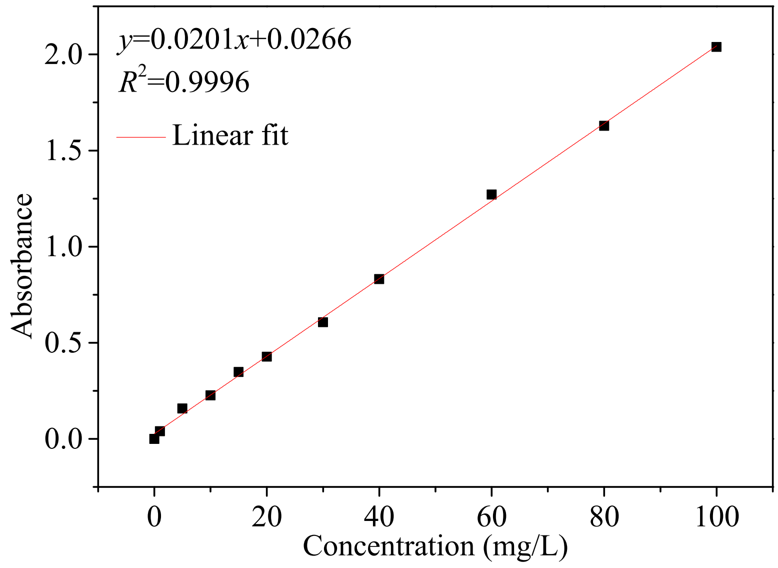

Figure S1. Standard calibration curve of DOX in DMSO.

A certain amount DOX was dissolved in DMSO to prepare the DOX-DMSO solution with a concentration of 100 mg/L. Then, the DOX-DMSO solution was diluted into a series of samples with gradient concentration ranging from 0–100 mg/L. The UV absorption intensity of the samples was recorded by a Lambda 950 UV-Vis spectrophotometer at a wavelength of 480 nm. Taking the UV absorption intensity and DOX concentration as the *x*-axis and *y*-axis, respectively, the standard calibration curve of DOX in DMSO could be obtained as shown in Figure S1.

S3 Dissipative Particle Dynamics (DPD) Simulation Methodology

DPD simulation is a stochastic coarse-grained simulation method suitable for complex multiphase systems, which make molecules dividing into sets of soft interacting beads. In DPD simulation, each bead is used to represent a group of atoms or a volume of fluid which is macroscopically small but still large on the atomistic scale. It can be used to investigate polymer systems combining the bead-spring model with the one described. All beads comply with the Newton's equations of motion (Equation S1 and Equation S2).

$$\frac{dr_i}{dt} = v_i \qquad \text{(S1)}$$

$$m_i \frac{dv_i}{dt} = f_i \qquad \text{(S2)}$$

where r_i, v_i, m_i and f_i are the position vector, velocity, mass and total force of particle i, respectively.

For simplicity, the masses of all beads are set to 1 DPD units. The force between each pair of beads is the sum of a conservative force (F_{ij}^C), a dissipative force (F_{ij}^D), and a random force (F_{ij}^R). The conservation force for non-bonded particles is defined by soft repulsion. The dissipative force corresponds to a frictional force that depends both on the position and relative velocities of the beads. The random force is a random interaction between a bead i and its neighbor bead j. All forces vanish beyond a certain cutoff radius, r_c, whose value is usually set to be 1 DPD unit of length in simulation. The three forces are given by Equation (S3), Equation (S4) and Equation (S5).

$$F_{ij}^C = \begin{cases} a_{ij}(1-r_{ij})\hat{r}_{ij} & (r_{ij} \prec 1) \\ 0 & (r_{ij} \succ 1) \end{cases} \qquad \text{(S3)}$$

$$F_{ij}^D = -\frac{\sigma^2(\omega(r_{ij}))^2}{2kT}(\hat{r}_{ij} \cdot v_{ij})\hat{r}_{ij} \qquad \text{(S4)}$$

$$F_{ij}^R = -\frac{\sigma(r_{ij})\hat{r}_{ij} \cdot \zeta}{\sqrt{\delta_t}} \qquad \text{(S5)}$$

Where $r_{ij}=r_i-r_j$, $\hat{r}_{ij}=r_{ij}/\left|\hat{r}_{ij}\right|$, $v_{ij}=v_i-v_j$, k is the Boltzman constant, T is the system temperature, σ is the noise strength, ζ denotes the randomly fluctuating variable with zero mean and unit variance, δ_t is time step of simulation, a_{ij} is the maximum repulsion between beads i and j.

In the DPD simulation, as shown in Figure S2, the polymers were divided into seven types of beads, including Ad (red), LA (blue), GA (indigo), MAA (pink), DEA (brown), TriAZO (purple) and mPEG (light blue). At the same time, the DOX (orange) was separated into three DOX1 beads, one DOX2 bead and one DOX3 bead, and the Water bead (yellow) consisted of seven water molecules. In the acidic environment, the protonated DEA and DOX3 beads were represented as DEAH and DOX3H beads, respectively. According to the Equation (S6) and Equation (S7), the

interaction parameter (a_{ij}) was calculated and the results were listed in Table S1. Typically, ΔE_{mix} which represented the mixing energy of the binary components could be obtained through simulation using Materials Studio 8.0 software. A 200 × 200 × 200 Å^3 cubic simulation box with a periodic boundary condition was utilized in all directions. The radius, volume and average mass of TriAZO bead were 3.4 Å, 163 Å^3 and 68 amu, respectively, while the radius, volume and average mass of the other beads were 3.7 Å, 213 Å^3 and 126 amu, respectively. Cutoff radius (r_c) is usually set to be 1 unit of length in simulations, and when the density of the beads was 3, the r_c of TriAZO bead and the other beads were 7.8 Å and 8.6 Å, respectively. The force field in the simulations of Amorphous Cell was COMPASS. The total simulation steps and integral time step were 200000 and 0.05 ns, respectively, and the spring constant was installed to 4.0 in our work.

$$X_{ij} = \frac{\Delta E_{mix} V_r}{RT\varphi_i \varphi_j V} \quad \text{(S6)}$$

$$a_{ij} = a_{ii} + 3.27 X_{ij} \quad \text{(S7)}$$

where χ_{ij} is Flory-Huggins parameters, ΔE_{mix} is mixing energy of the binary components, V_r and V are the reference volume and total volume, R is the gas constant and T *is* temperature, φ_i (φ_j) are the volume fractions of bead i (j), a_{ii} is repulsive parameter of beads i which is equal to 25, a_{ij} is repulsive parameter between bead i and j.

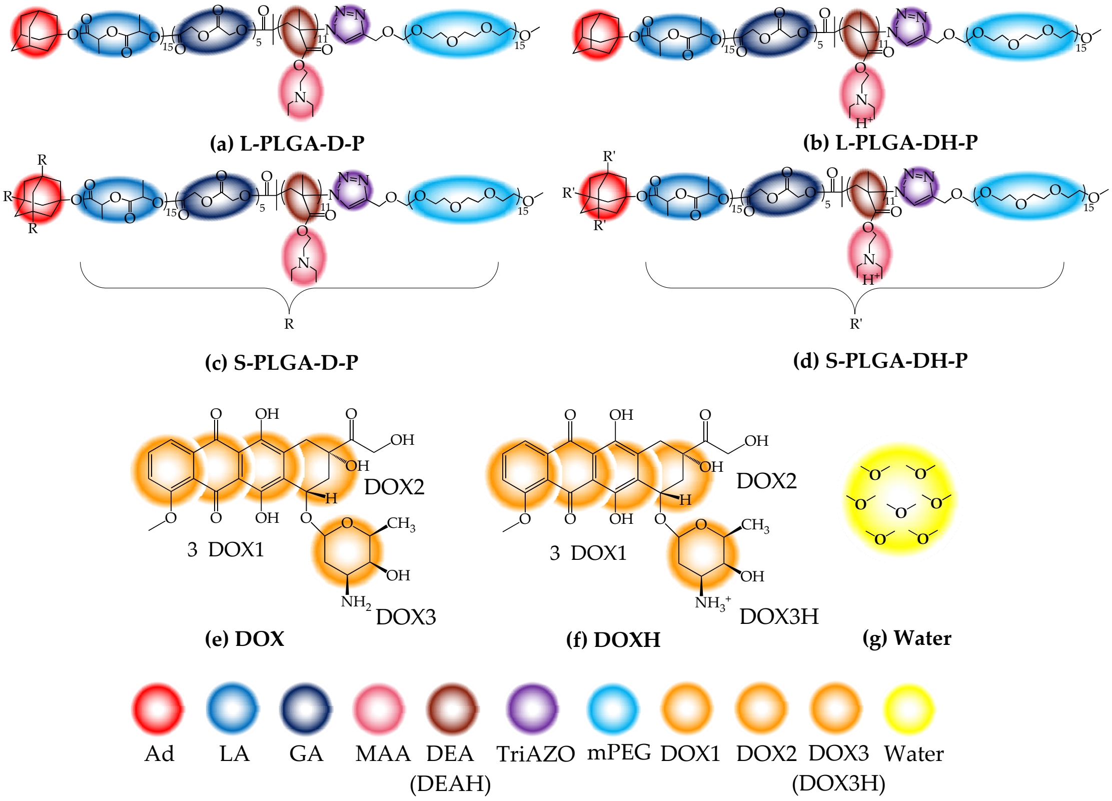

Figure S2. Coarse grain models of (**a**) L-PLGA-D-P, (**b**) L-PLGA-DH-P, (**c**) S-PLGA-D-P, (**d**) S-PLGA-DH-P, (**e**) DOX, (**f**) DOXH, (**g**) Water.

Table S1. Interaction parameters a_{ij} between different beads used in DPD simulation.

a_{ij}	Ad	LA	GA	MAA	DEA	DEAH	TriAZO	mPEG	DOX1	DOX2	DOX3	DOX3H	Water
Ad	25.0												
LA	25.4	25.0											

GA	27.0	26.1	25.0										
MAA	27.5	27.3	25.3	25.0									
DEA	26.1	25.6	25.0	25.4	25.0								
DEAH	99.9	108	142	51.4	—	25.0							
TriAZO	26.7	26.4	25.0	25.1	25.1	49.5	25.0						
mPEG	37.8	41.5	33.5	28.5	31.7	23.0	29.2	25.0					
DOX1	29.2	30.3	26.8	25.4	26.4	34.6	25.6	26.1	25.0				
DOX2	31.7	32.5	27.8	25.8	27.5	39.5	26.2	26.2	25.0	25.0			
DOX3	36.0	39.3	32.0	27.7	30.6	—	28.4	25.0	25.8	25.8	25.0		
DOX3H	159	115	127	101	—	19.8	97.2	21.1	22.7	24.3	—	25.0	
Water	173	161	104	129	154	23.5	128	26.1	47.0	38.2	101	12.7	25.0

DPD simulation of drug loading behavior for polymeric micelles was carried out, and the results were shown in Figure S3.

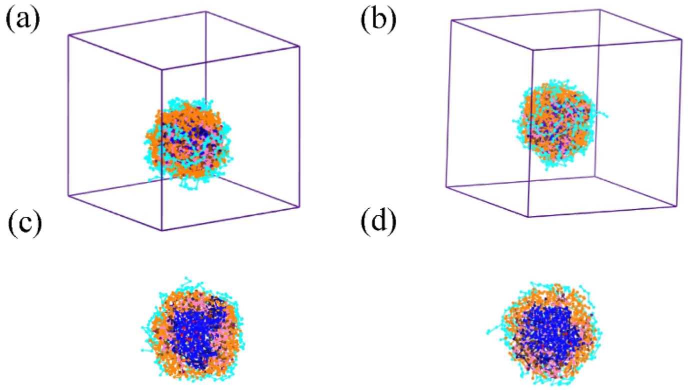

Figure S3. DPD edquilibrium states of DOX-loaded L-PLGA-D-P micelles (**a**) and DOX-loaded S-PLGA-D-P micelles (**b**). Cross-section views of DOX-loaded L-PLGA-D-P micelles (**c**) and DOX-loaded S-PLGA-D-P micelles (**d**). The beads of Ad, LA, GA, MAA, DEA, TriAZO, mPEG and DOX were represented in red, blue, indigo, pink, brown, purple, light blue and orange, respectively.

S4 ^{1}H NMR Spectra of as-synthesized Polymers and their Precursors

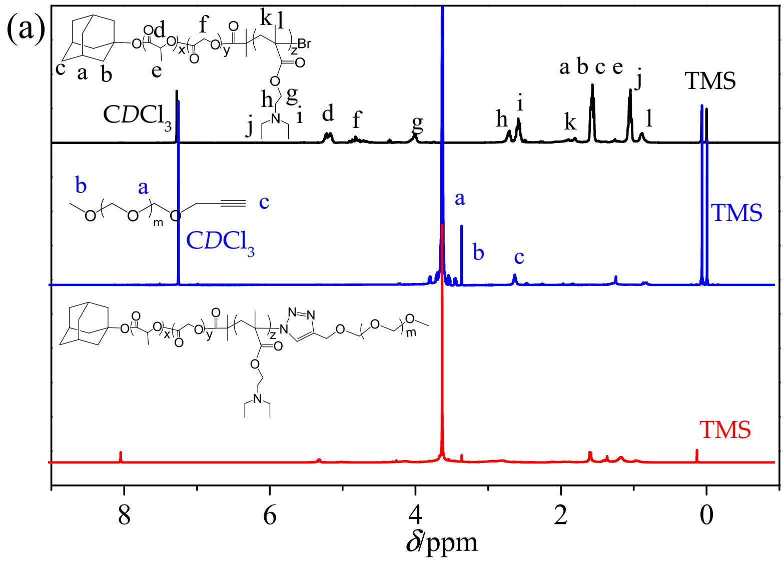

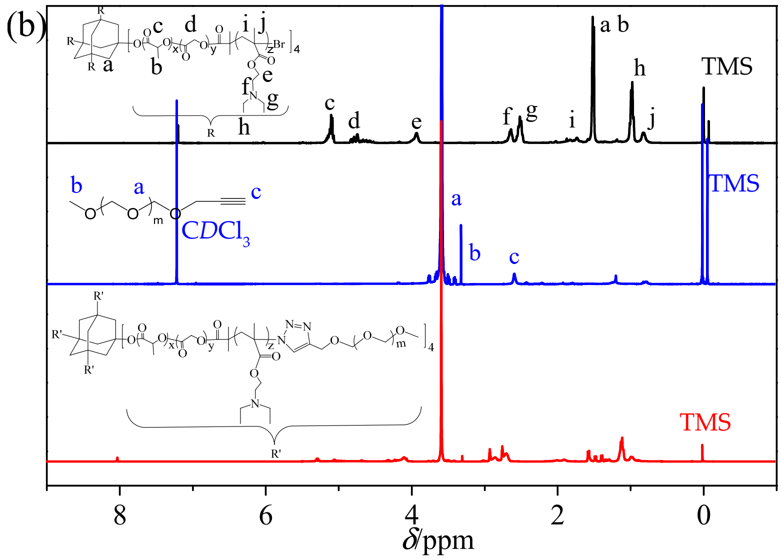

Figure S4. ^{1}H NMR spectra of polymers L-PLGA-D-P (**a**), S-PLGA-D-P (**b**) and the CuAAC precursors.

The ^{1}H NMR spectra of the CuAAC precursors and as-prepared polymers were recorded using deuterated chloroform (C*D*Cl$_3$) and deuterated *N,N'*-dimethylformamide (DMF-*d$_7$*) as the deuterated solvents. For the ^{1}H NMR spectrum of Ad-P(LA-*co*-GA)-*b*-PDEAEMA (Figure S4a), the signal at 1.68 ppm was ascribed to the resonance of the protons belonging to adamantane core, and the resonant signals at 1.68 ppm, 4.90 ppm and 5.17 ppm corresponded to –CH_3, –CH_2-, –CH– belonging to P(LA-*co*-GA) backbone, respectively. In addition, the signals of –CH_2CH_2– in PDEAEMA block adjacent to oxygen and nitrogen atoms were found at 2.71 ppm and 4.00 ppm, whereas the signals of –CH_2– and –CH_3 at the end of PDEAEMA block were found at 1.08 ppm and 2.61 ppm, respectively. For the ^{1}H NMR spectrum of alkynyl-mPEG, the strong resonant signals at 3.58 ppm and 3.29 ppm corresponded to –CH_2– and –CH_3 in mPEG backbone, respectively, while the signal of proton for the terminal ethynyl was found at 2.68 ppm. For the ^{1}H NMR spectrum of Ad-[P(LA-*co*-GA)-*b*-PDEAEMA]$_4$ (Figure S4b), the signals for the corresponding protons were similar to those found in the ^{1}H NMR spectrum of Ad-P(LA-*co*-GA)-*b*-PDEAEMA. ^{1}H NMR for Ad-[P(LA-*co*-GA)-*b*-PDEAEMA]$_4$, δ 0.99 ppm (h), δ 1.74 ppm (a + b), δ 2.54 ppm (g), δ 2.64 ppm (f), δ 3.94 ppm (e), δ 4.84 ppm (d), δ 5.10 ppm (c).

S5 FTIR Spectra of as-synthesized Polymers and their Precursors

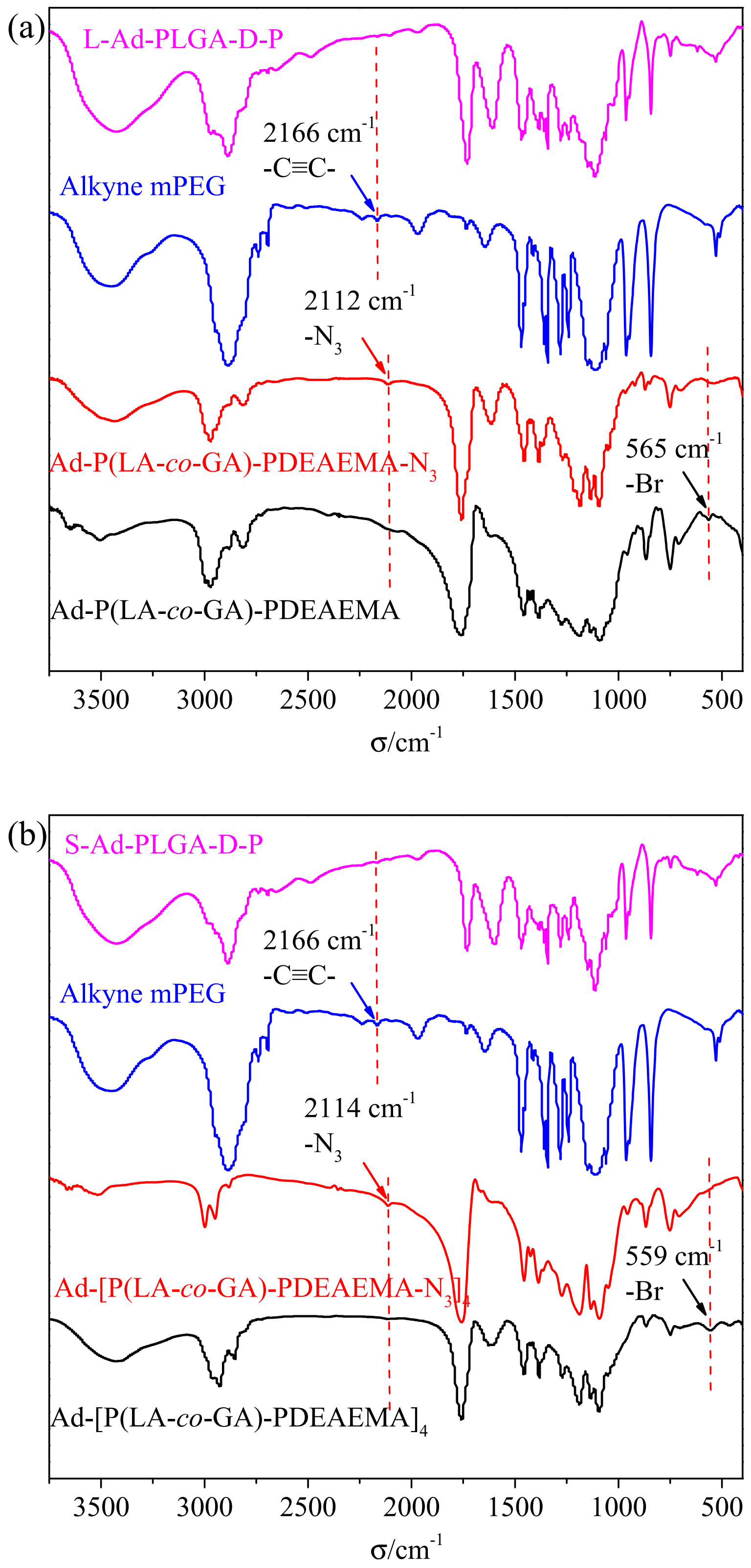

Figure S5. FTIR spectra of polymers L-PLGA-D-P (**a**), S-PLGA-D-P (**b**) and their CuAAC precursors.

S6 GPC Results of the Polymers and their Precursors

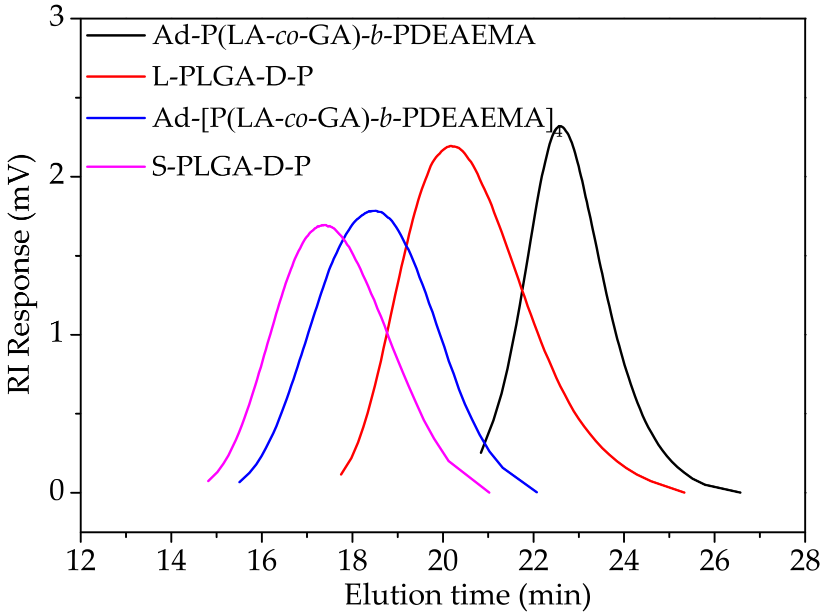

Figure S6. GPC traces of L-PLGA-D-P, S-PLGA-D-P and their precursors.

S7 ¹H NMR Spectra of Polymers in Deuterated Water (D_2O)

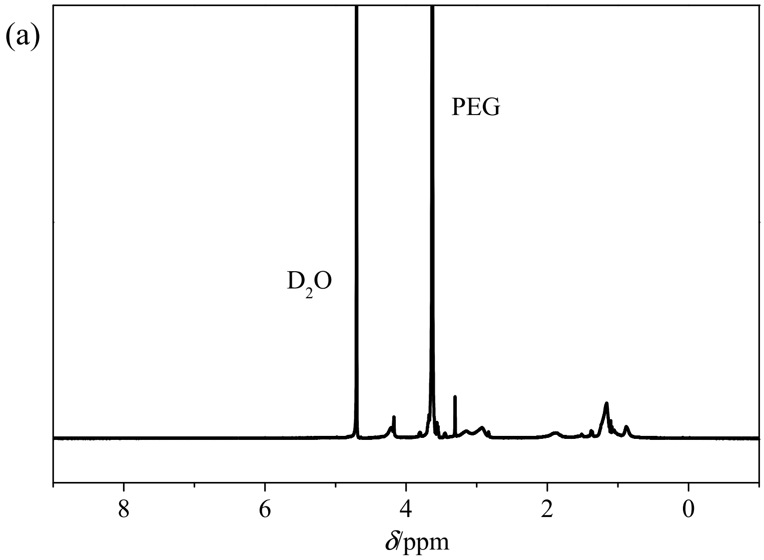

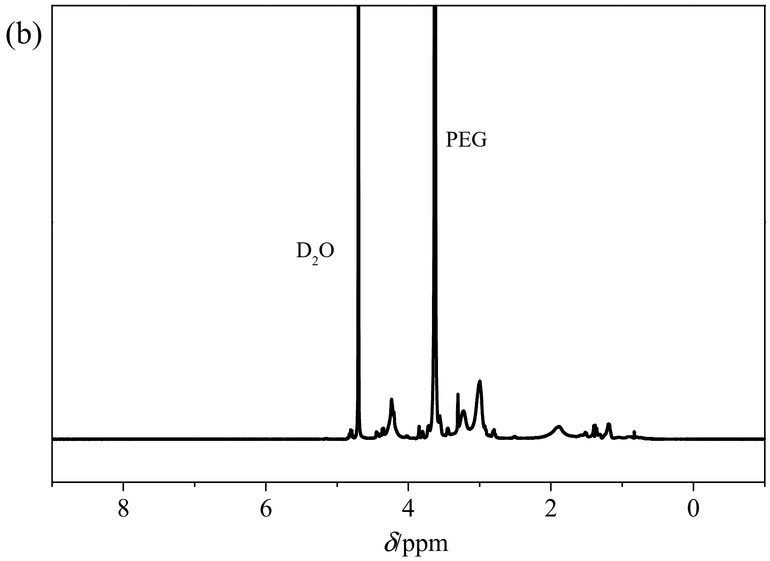

Figure S7. ^{1}H NMR spectra of L-PLGA-D-P (**a**) and S-PLGA-D-P (**b**) in D_2O.

Figure S7 presented the ^{1}H NMR spectra of L-PLGA-D-P (a) and S-PLGA-D-P (b) in D_2O. The $-CH_2-$ characteristics signal of hydrophilic PEG segment was found at 3.58 ppm.

S8 Particle Size Distribution of Polymeric Micelles determined by DLS with different pH Values.

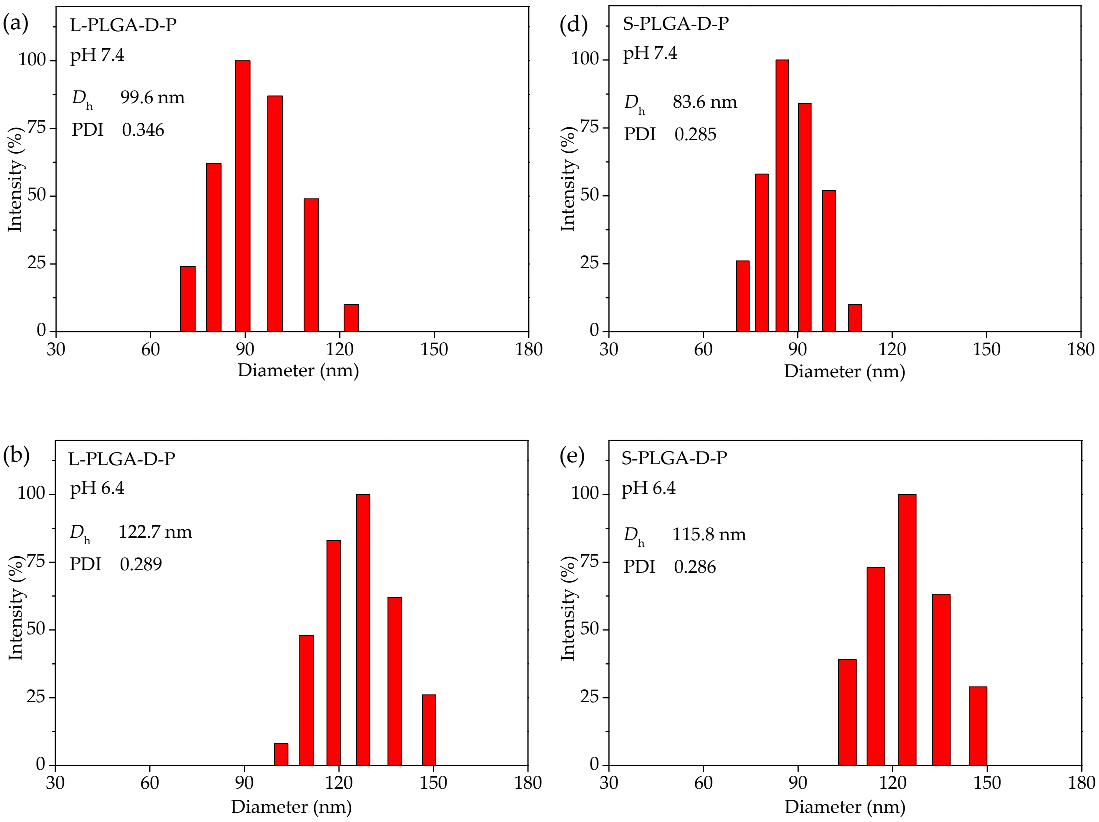

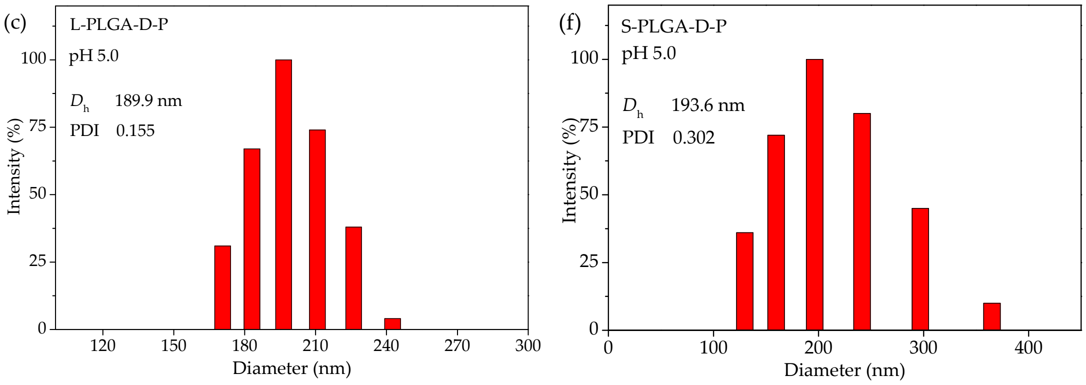

Figure S8. Particle size distribution of L-PLGA-D-P determined by DLS after 12 h incubation, (**a**) pH 7.4, (**b**) pH 6.4, (**c**) pH 5.0. Particle size distribution of S-PLGA-D-P determined by DLS after 12 h incubation, (**d**) pH 7.4, (**e**) pH 6.4, (**f**) pH 5.0.

The particle sizes of polymeric micelles were measured by DLS at room temperature in phosphate buffer saline medium (PBS, pH 7.4 and 6.4) or acetate buffer medium (pH 5.0). The particle sizes of L-PLGA-D-P were 99.6 nm, 122.7 nm and 189.9 nm at pH 7.4, 6.4 and 5.0, respectively. The particle sizes of S-PLGA-D-P were 83.6 nm, 115.8 nm and 193.6 nm at pH 7.4, 6.4 and 5.0, respectively.

S9 IC_{50} Values of Polymeric Micelles, DOX-loaded Micelles and Free DOX

Table S2. IC_{50} values of blank micelles, DOX-loaded micelles and free DOX.

Samples	[a] IC_{50} (μg/mL)	
	**NIH-3T3**	**MCF-7**
L-PLGA-D-P	>300	>300
DOX@L-PLGA-D-P	>300	14.600
S-PLGA-D-P	>300	>300
DOX@S-PLGA-D-P	>300	9.535
DOX	<1.23	0.082

[a] Half maximal inhibitory concentration.
